# Obstetric Anal Sphincter Injuries (OASIs) in Israel: A Review of the Incidence and Risk Factors

**DOI:** 10.5041/RMMJ.10295

**Published:** 2017-04-28

**Authors:** Shimon Ginath, Yossi Mizrachi, Jacob Bar, Alexander Condrea, Michal Kovo

**Affiliations:** Department of Obstetrics and Gynecology, the Edith Wolfson Medical Center, Holon, Israel, affiliated to the Sackler Faculty of Medicine, Tel Aviv University, Tel Aviv, Israel

**Keywords:** Israel, OASIs, workshop

## Abstract

Obstetric anal sphincter injuries (OASIs) following vaginal deliveries are the main reason for subsequent development of anal incontinence in women. The diagnosis of such tears is crucial for treating and preventing such a grave sequela. The reported rate of OASIs in Israel was between 0.1% and 0.6%, out of all vaginal births, which is 10-fold lower than that reported in Europe and the United States. Structured hands-on training in repair of OASIs in seven medical centers in Israel significantly increased the detection rate of third-degree perineal tears. The implementation of such programs is crucial for increasing awareness and detection rates of OASIs following vaginal deliveries.

## INTRODUCTION

Perineal tears following childbirth are longitudinal, extending from the vulva, and reach and involve the anal sphincter.^1^ It is of major importance to recognize the full extent of the damage, since repair must be meticulous in order to avoid preventable complications. Perineal tears are classified into four grades according to their extent: 2

1. First-degree tears include injury to the skin only.
2. Second-degree tears include injury to the perineum involving perineal muscles but not the anal sphincter.
3. Third-degree tears include injury to perineum involving the anal sphincter complex. Third-degree tears are further divided into (i) less than 50% of the external anal sphincter thickness torn, (ii) more than 50% of the external anal sphincter thickness torn, and (iii) internal anal sphincter torn.
4. Fourth-degree tears include injury to the peri-neum involving the anal sphincter complex and anal epithelium.

This classification has been adopted by the International Consultation on Incontinence and the Royal College of Obstetricians and Gynaecologists (RCOG).^3^

Severe perineal tears during childbirth (third- and fourth-degree) are often referred to as obstetric anal sphincter injuries (OASIs). These tears are associated with high rates of early complications, such as pain and infection, as well as late complications, such as chronic pain, dyspareunia, fecal incontinence, urinary incontinence, pelvic organ prolapse, fistula formation, and psychological problems.^4^ Obstetric anal sphincter injuries are considered the most important risk factors for fecal incontinence, as a result of mechanical disruption of the anal sphincter muscles and/or damage to the nerves innervating these muscles.^5^

The reported prevalence of OASIs varies widely, ranging from 0.1% to 19% among different populations, depending on parity, type of episiotomy used, rates of operative vaginal delivery, misclassification, and misdiagnosis.^6^ The reported European and American rates of OASIs are 2%–6% of all vaginal singleton deliveries.^7^–^9^ Based on meta-analysis of data from 22 studies that include 651,934 women, of whom 15,366 (2.4%) had severe lacerations, the main risk factors for severe perineal tears were: larger infants (mean difference, 192.88 g; 95% CI, 139.80–245.96 g), episiotomy (OR, 3.82; 95% CI, 1.96–7.42), operative vaginal delivery (OR, 5.10; 95% CI, 3.33–7.83), epidural anesthesia (OR, 1.95; 95% CI, 1.63–2.32), labor induction (OR, 1.08; 95% CI, 1.02–1.14), labor augmentation (OR, 1.95; 95% CI, 1.56–2.44), primiparity (OR, 3.24; 95% CI, 2.20–4.76), Asian ethnicity (OR, 2.74; 95% CI, 1.31–5.72), and persistent occiput posterior position (OR, 3.09; 95% CI, 1.81–5.29).^10^,^11^ In Israel, according to several reports, the reported incidence of severe perineal tears is lower, compared to other reports.

The aim of this study is to report the incidence and risk factors of severe perineal tears as published in peer-reviewed studies from Israel.

## MATERIAL AND METHODS

A systematic computerized search of the literature, from January 2000 to December 2016, was performed in PubMed/MEDLINE to identify relevant articles to be included in this review. All pertinent articles were examined, and their reference lists were systematically reviewed to identify other studies for potential inclusion in this article. The following key words and medical subject heading (MeSH) terms were used: “Obstetric anal sphincter injuries,” “OASI,” “severe perineal tears,” and “third- and fourth-degree perineal tears” in combination with “Israel.” The studies included in our analysis met all the following criteria: incidence and risk factors of OASI were presented, in peer-reviewed papers, published in the English language. No institutional review board approval was required because only published, de-identified data were analyzed.

## RESULTS

Eight papers were found after the search describing the incidence and risk factors of OASIs in Israel. The reported rates of OASIs in Israel are 0.1%–0.6% (Table 1).^4^,^6^,^12^–^17^ The main risk factors are summarized in Table 2 and include instrumental deliveries, primiparity, fetal macrosomia, persistent occipito-posterior position, precipitate labor, prolonged second stage of labor, lower midwife experience, and vaginal birth after cesarean section.

**Table 1:** Incidences of OASIs in Israel.

Study	Study Period	Medical Center	Events	Incidence
Sheiner et al.(2005)^16^ *	1988–1999	Soroka	79/98,524	0.08%
Groutz et al.(2011)^13^ *	2005–2009	Sourasky	96/38,252	0.25%
Zafran & Salim (2012)^17^ *	2004–2008	Emek	62/15,705	0.39%
Melamed et al. (2013)^15^†	1999–2011	Rabin	356/58,937	0.60%
Loewenberg-Weisband et al.(2014)^14^*	2006–2011	Shaarei Zedek	214/61,308	0.35%
Garmi et al.(2016)^12^*	2004–2012	Emek	111/28,896	0.39%
Krissi et al.(2016)^6^†	2010–2013	Rabin	74/20,484	0.36%
Mizrachi et al.(2017)^4^‡	2011–2015	Wolfson	51/15,146	0.34%
**Total**			**1,045/337,252**	**0.31%**

* Singleton, term (≥37 weeks’ gestation), vertex presentation, and vaginal delivery.

† Singleton, viable infant (≥24 weeks’ gestation, ≥500 g), vertex presentation, and vaginal delivery.

‡ Singleton, term (≥37 weeks’ gestation), vertex presentation, and spontaneous vaginal delivery.

**Table 2:** Independent Risk Factors for OASIs in Israel.

Study	Risk Factors
Sheiner et al. (2005)^16^	Forceps delivery (OR, 26.7; 95% CI, 8.0–88.5) Vacuum extraction (OR, 8.2; 95% CI, 4.7–14.5) Fetal macrosomia (>4,000 g) (OR, 2.5; 95% CI, 1.2–4.9)
Groutz et al. (2011)^13^	Asian ethnicity (OR, 8.9; 95% CI, 4.2–18.9) Vacuum delivery (OR, 2.7; 95% CI, 1.6–4.6) Primiparity (OR, 2.4; 95% CI, 1.5–3.7) Persistent occipito-posterior position (OR, 2.1; 95% CI, 1–4.5) Birthweight ≥4,000 g (OR, 1.001; 95% CI, 1–1.001)
Zafran & Salim (2012)^17^	Primiparity (OR, 11.75; 95% CI, 3.10–44.60) Vacuum extraction (OR, 4.21; 95% CI, 1.31–13.53)
Melamed et al. (2013)^15^	Forceps delivery (OR, 5.5; 95% CI, 3.9–7.8) Precipitate labor (OR, 5.2; 95% CI, 2.9–2.9) Persistent occiput-posterior position (OR, 2.6; 95% CI, 1.6–4.3) Vacuum extraction (OR, 1.9; 95%CI, 1.4–2.6) Large for gestational age (>90th percentile) (OR, 1.5; 95% CI, 1.1–2.0) Gestational age >40 weeks (OR, 1.4; 95% CI, 1.1–1.7)
Loewenberg-Weisband et al. (2014)^14^	Primiparity (OR, 3.19; 95% CI, 2.23–4.55) Instrumental delivery (OR, 1.82; 95% CI, 1.25–2.65) Prolonged second stage of labor (OR, 1.77; 95% CI, 1.19–2.61) Episiotomy (OR, 1.69; 95% CI, 1.18–2.40)
Garmi et al. (2016)^12^	Vaginal birth after cesarean section (OR, 13.6; 95% CI, 4.7–39.3) Primiparity (OR, 7.6; 95% CI, 3.5–16.3) Prolonged second stage of labor (OR, 1.5; 95% CI, 1.1–2.1)
Krissi et al. (2016)^6^	Type 2 diabetes mellitus (OR, 24.5; 95% CI, 2.55–236.40) Severe preeclampsia (OR, 9.53; 95% CI, 1.17–77.55) Vaginal birth after cesarean section (OR, 3.65; 95% CI, 1.49–9.12) Higher neonatal birthweight (OR, 1.01; 95% CI, 1.01–1.02)
Mizrachi et al. (2017)^4^	Nulliparity (OR, 6.08; 95% CI, 2.30–16.02) Fetal macrosomia (OR, 4.17; 95% CI 1.36–12.75)

Sheiner et al.^16^ reported an incidence of 0.1% for third-degree perineal tears in singleton, term vaginal deliveries. On multivariate analysis, independent risk factors for third-degree perineal tears were fetal macrosomia (>4,000 g) (OR, 2.5; 95% CI, 1.2–4.9), vacuum extraction (OR, 8.2; 95% CI, 4.7–14.5), and forceps delivery (OR, 26.7; 95% CI, 8.0–88.5(.

Groutz et al.^13^ reported an incidence of 0.25% for third- and fourth-degree perineal tears in singleton, term, vertex vaginal deliveries. Independent risk factors for perineal tears were Asian ethnicity (OR, 8.9; 95% CI, 4.2–18.9), primiparity (OR, 2.4; 95% CI, 1.5–3.7), persistent occipito-posterior position of the head (OR, 2.1; 95% CI, 1–4.5), vacuum delivery (OR, 2.7; 95% CI, 1.6–4.6), and birthweight ≥4,000 g (OR, 1.001; 95% CI, 1–1.001).

Zafran and Salim^17^ reported an incidence of 0.4% for OASIs in singleton, term, vertex vaginal deliveries. Independent risk factors for perineal tears were vacuum extraction (OR, 4.21; 95% CI, 1.31–13.53) and primiparity (OR, 11.75; 95% CI, 3.10–44.60).

Melamed et al.^15^ reported an incidence of 0.6% for third- and fourth-degree perineal tears in single-ton, viable (≥24 weeks’ gestation, ≥500 g), vertex, vaginal deliveries. Independent predictors of OASIs were forceps delivery (OR, 5.5; 95% CI, 3.9–7.8), precipitate labor (OR, 5.2; 95% CI, 2.9–9.2), persistent occiput posterior position (OR, 2.6; 95% CI, 1.6–4.3), vacuum extraction (OR, 1.9; 95% CI, 1.4–2.6), large-for-gestational-age neonates (>90th percentile) (OR, 1.5; 95% CI, 1.1–2.0), and gestational age >40 weeks (OR, 1.4; 95% CI, 1.1–1.7).

Loewenberg-Weisband et al.^14^ reported an incidence of 0.35% for severe (third- and fourth-degree) perineal tears, in singleton, term, vaginal deliveries. Independent predictors of OASIs were instrumental delivery (OR, 1.82; 95% CI, 1.25–2.65), prolonged second stage of labor (OR, 1.77; 95% CI, 1.19–2.61), primiparity (OR,3.19; 95% CI, 2.23–4.55), and episiotomy (OR, 1.69; 95% CI, 1.18–2.40).

Garmi et al.^12^ reported an incidence of 0.4% for OASIs in singleton, term, vertex vaginal deliveries. Independent risk factors for OASIs were primiparity (OR, 7.6; 95% CI, 3.5–16.3), vaginal birth after previous cesarean section (OR, 13.6; 95% CI, 4.7–39.3), and prolonged second stage of labor (OR, 1.5; 95% CI, 1.1–2.1). For every 1-hour increase in the length of the second stage, the odds for OASIs increased 1.5 times.

Krissi et al.^6^ reported an incidence of 0.6% for third- and fourth-degree perineal tears in singleton, viable (≥24 weeks’ gestation, ≥500 g), vertex, vaginal deliveries. Factors independently associated with an increased risk for OASIs were vaginal birth after cesarean section (OR, 3.65; 95% CI, 1.49–9.12), higher neonatal birthweight (OR, 1.01; 95% CI, 1.01–1.02), severe preeclampsia (OR, 9.53; 95% CI, 1.17–77.55), and type 2 diabetes mellitus (OR, 24.5; 95% CI, 2.55–236.40). Factors that were inde-pendently associated with a decreased risk for OASIs were parity (OR, 0.37; 95% CI, 0.25–0.54) and spontaneous vaginal delivery (OR, 0.43, 95% CI, 0.26–0.71).

Mizrachi et al.^4^ reported an incidence of 0.3% for severe perineal tears (third-and fourth-degree) in singleton, term, vertex, spontaneous vaginal deliveries. Independent risk factors for OASIs were nulliparity (OR, 6.08; 95% CI, 2.30–16.02) and fetal macrosomia (OR, 4.17; 95% CI 1.36–12.75). Interestingly, midwife experience was also independently associated with a lower rate of severe perineal tears (OR, 0.95; 95% CI 0.91–0.99). Moreover, each additional year of experience was associated with a 4.7% decrease in the risk of severe perineal tears.

## DISCUSSION

The reported European and American rates of OASIs are 2%–6% of all vaginal singleton deliveries.^7^–^9^ In Israel, according the reviewed papers, the reported incidence of severe perineal tears is 0.1%–0.6%, ten times lower compared to other reports. However, the risk factors are similar.

The very low rate of OASIs in Israel may be related to avoidance of midline episiotomy and almost extinct use of forceps deliveries. It can be explained by proper technique of perineal protection at the time of delivery of the head.^18^ Incorporation of a manual perineal protection technique at the crowning of the fetal head decreased significantly the incidence of OASIs; the technique consisted of four components: (1) slowing the delivery of the baby’s head with one hand, (2) supporting the perineum with the other hand and squeezing with fingers (first and second) from the perineum lateral parts towards the middle in order to lower the pressure in the middle posterior perineum, (3) asking the delivering woman not to push, and (4) performing correct episiotomy only when indicated.^19^–^22^

The low OASI rate can be explained also by under-diagnosis probably due to low awareness of such damage. In order to increase the detection rate and awareness of OASIs, among the residents and physicians, the Israeli Society of Urogynecology and Pelvic Floor Surgery initiated a hands-on workshop on the diagnosis and repair of third- and fourth-degree perineal tears, starting February 2011 till now. Andrews et al.^23^ demonstrated that structured hands-on training and repair of OASIs increased the detection rate of OASIs mainly because of a higher awareness of such damage.

The structure of the hands-on workshop in Israel resembles the workshop organized by Mr Abdul Sultan and Miss Ranee Thakar at the International Urogynecological Association (IUGA) annual conferences. The workshop in Israel consists of series of lectures and videos demonstrating proper identification techniques of OASIs, followed by a hands-on training session on cadaveric anal sphincters from pigs. Participants were all physicians (seniors and residents) attending the delivery ward at the respective medical center. In seven medical centers, data were collected calculating the rate of OASIs one year before and one year following the workshop. The results were presented as an abstract at an IUGA meeting.^24^ Inclusion criteria were singleton pregnancy, vertex presentation, and vaginal delivery. A total of 39,920 consecutive deliveries occurred during the year prior to the workshop, and 41,211 delivered occurred during the year following the workshop in the reviewed centers. Third- or fourth-degree perineal tears occurred in 153 women (0.38%) before the workshop, and in 197 (0.48%) following the workshop, an increase of 25% (*P*=0.045). The increase in diagnosis was most significant in third-degree perineal tears: 137 women (0.34%) before the workshop and 183 (0.44%) following the workshop, an increase of 29% (*P*=0.025).

In conclusion, the rate of severe perineal lacerations during vaginal deliveries in Israel is much lower than the rate in the United States and Europe. This, in part, may be the result of misdiagnosis. Implementation of training programs may increase the diagnosis rate, and subsequently more women will receive a proper treatment, in order to improve maternal long-term outcomes.

## Abbreviations

OASIs: obstetric anal sphincter injuries
